# miR-223 promotes colon cancer by directly targeting p120 catenin

**DOI:** 10.18632/oncotarget.19541

**Published:** 2017-07-25

**Authors:** Liwei Liu, Chao Zhang, Xiyu Li, Wenjia Sun, Shenghui Qin, Lingzhi Qin, Xi Wang

**Affiliations:** ^1^ Institute of Pathology, Tongji Hospital, Tongji Medical College, Huazhong University of Science and Technology, Wuhan 430030, China

**Keywords:** colon cancer, miR-223, p120, RhoA, β-catenin

## Abstract

microRNA (miRNA) dysregulation is frequently observed in colon cancer. Previous studies found that miR-223 is upregulated in colon cancer and functions as an oncogene. Conversely, p120 is often downregulated or even absent in colon cancer, and is a likely tumor suppressor. The present study showed that increased miR-223 and decreased p120 levels are associated with colon cancer malignancy, and p120 expression is negatively correlated with miR-223 expression. A dual luciferase reporter assay showed that miR-223 directly targets p120. miR-223 upregulation in a colon cancer cell line upregulated c-Myc, cyclinD1, MMP7, and vimentin expression, downregulated E-cadherin, increased nuclear expression of β-catenin, and enhanced RhoA activation. We suggest miR-223 may promote colon cancer cell invasion and metastasis by downregulating p120, thereby reducing intercellular adhesion, promoting RhoA activity, and activating β-catenin signaling. Thus miR-223 functions as an oncogene in colon cancer and may be a potential diagnostic and therapeutic target for anti-colon cancer treatment.

## INTRODUCTION

Colon cancer is the third most common and deadly malignant tumor type in both men and women [1]. Recurrence and metastasis are the main causes of death in colon cancer patients. Tumor invasion involves complex gene regulatory events, including decreased adhesion between tumor cells, detachment of tumor cells from the primary site, and destruction of extracellular matrix barriers. Detached tumor cells enter the circulatory system and may form distant metastases [2, 3].

Adhesion junctions (AJs) are important for maintaining intercellular adhesion, and include the E-cadherin (E-cad)/p120 catenin (p120)/β-catenin/α-catenin complex. p120 released from AJs reduces intercellular adhesion through E-cad degradation and promotes cell migration by regulating Rho GTP activity [4–6]. p120 is a member of the catenin family that binds the highly conserved E-cad juxtamembrane domain, stabilizing E-cad at the membrane, and regulating E-cad endocytosis. p120 is reportedly downregulated or translocated in a variety of human tumors and is often associated with poor prognosis [7–12]. *p120* gene mutations are rare, suggesting that other mechanisms, such as transcriptional downregulation, epigenetic modification, or microRNA-mediated gene silencing, may downregulate p120 in tumors [13].

Many microRNAs (miRNAs) reportedly play roles in tumor development [14]. miRNAs are a class of small, non-coding RNAs approximately 18–25 nucleotides in length that post-transcriptionally regulate mRNA. miRNAs can directly combine with the target mRNA 3′ untranslated region (3′-UTR), inhibiting target mRNA translation or promoting its degradation [15]. miR-223 expression is increased in colon cancer patient serum and tumor tissues [16, 17], and is associated with malignant tumor behavior and poor prognosis [18, 19]. The present study confirmed that p120 is a miR-223 target gene, and assessed miR-223 tumor promotion mechanisms in LoVo cells. We suggest that miR-223 may be a new target for colon cancer early diagnosis and treatment.

## RESULTS

### p120 is downregulated in human colon cancer tissues

**Figure 1:**
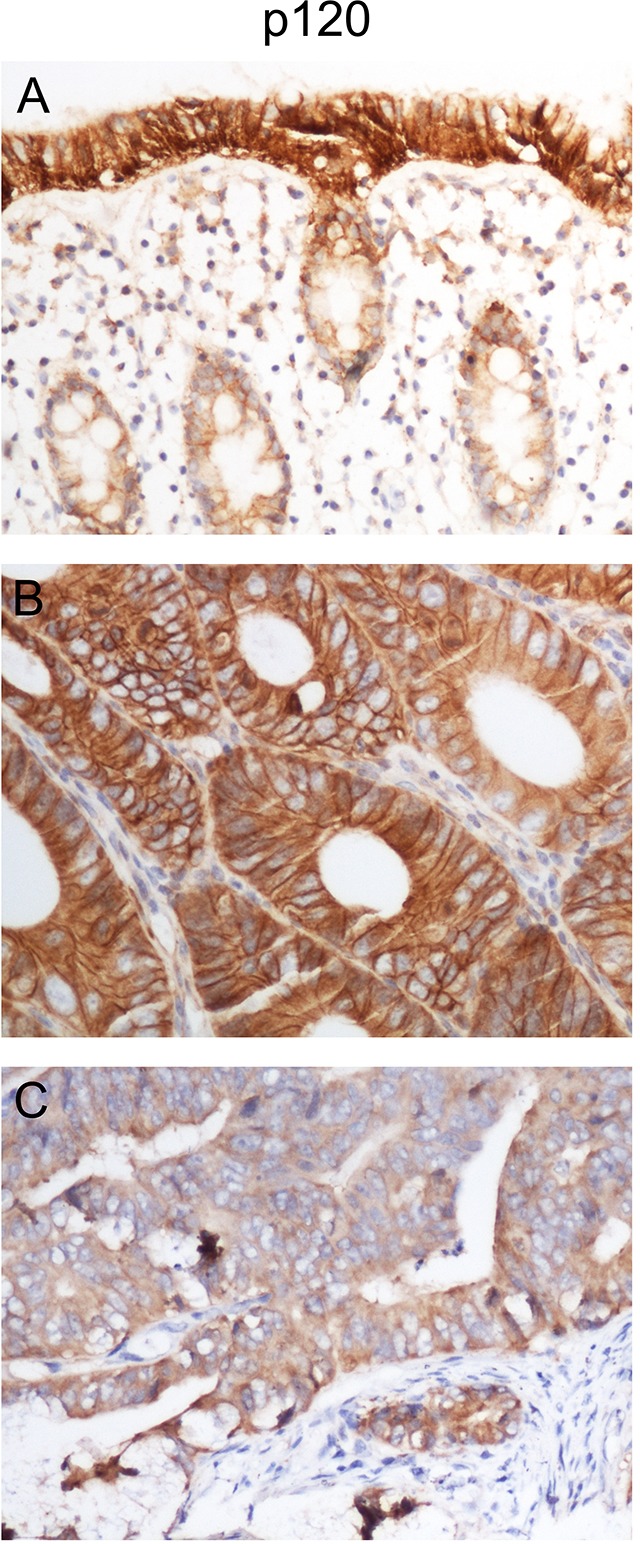
p120 is downregulated in human colon cancer tissues IHC staining was performed in 80 colon cancer tissues and 42 corresponding normal colon tissues. p120 was highly expressed in normal colonic tissues **(A)** and some colon cancer tissues **(B)**. p120 exhibited low expression in most colon cancer tissues **(C)**. Magnification, ×200.

In normal colonic tissues, p120 is uniformly expressed on epithelial cell membranes in a continuous linear pattern (Figure 1A). p120 expression was high in some colon cancer tissues (Figure 1B), but decreased or was even lost in most of these tissues (Figure 1C). Immunohistochemical (IHC) staining showed that the low expression rates of p120 in colon cancer and normal colon tissues were 71.25% and 0% (P<0.05), respectively (Table 1).

**Table 1:** p120 expression in normal colon and colon cancer tissues

	Low expression	High expression	χ^2^	*P*
**Colon cancer tissues**	57	23	56.167	0.000*
**Normal colon tissues**	0	42		

*P<0.05.

The low expression rates of p120 in poorly differentiated, moderately differentiated, and well differentiated tumor tissues were 91.67%, 43.48%, and 33.33% (P<0.05), respectively. The low expression rates of p120 in tissues with and without lymph node metastasis were 85.29% and 60.87% (P<0.05), respectively. The low expression rates of p120 in patients with Dukes stage C-D and stage A-B disease were 85.71% and 58.70% (P<0.05), respectively. When the transverse colon was defined as the right side colon, the low expression rates of p120 in patients with left side colon cancer and right side colon cancer were 57.14% and 78.85% (P=0.041), respectively. p120 expression was not associated with patient age, gender, tumor size, depth of tumor invasion, or other characteristics (P>0.05) (Table 2).

**Table 2:** p120 expression in colon cancer and its relationship with clinical and pathological features

Clinical parameters	Low expression	High expression	χ^2^	*P*
**Age (years)^a^**				
<58	30	11	0.151	0.697
≥58	27	12		
**Sex**				
Male	36	11	1.59	0.207
Female	21	12		
**Tumor size^b^**				
<5 cm	22	12	1.236	0.266
≥5 cm	35	11		
**Tumor location**				
Left	16	12	4.185	0.041*
Right	41	11		
**Differentiation grade**				
Well	3	6	24.744	0.000*
Moderately	10	13		
Poor	44	4		
**Invasive depth**				
T1-T3	29	13	0.209	0.647
T4	28	10		
**Lymph node metastasis**				
N0	28	18	5.693	0.017*
N1-N2	29	5		
**Dukes stage**				
A-B	27	18	6.355	0.012*
C-D	30	5		

^a^Age was grouped based on median age.

^b^Tumor size was grouped based on median tumor size.

*P<0.05.

### miR-223 is upregulated in human colon cancer tissues

**Figure 2:**
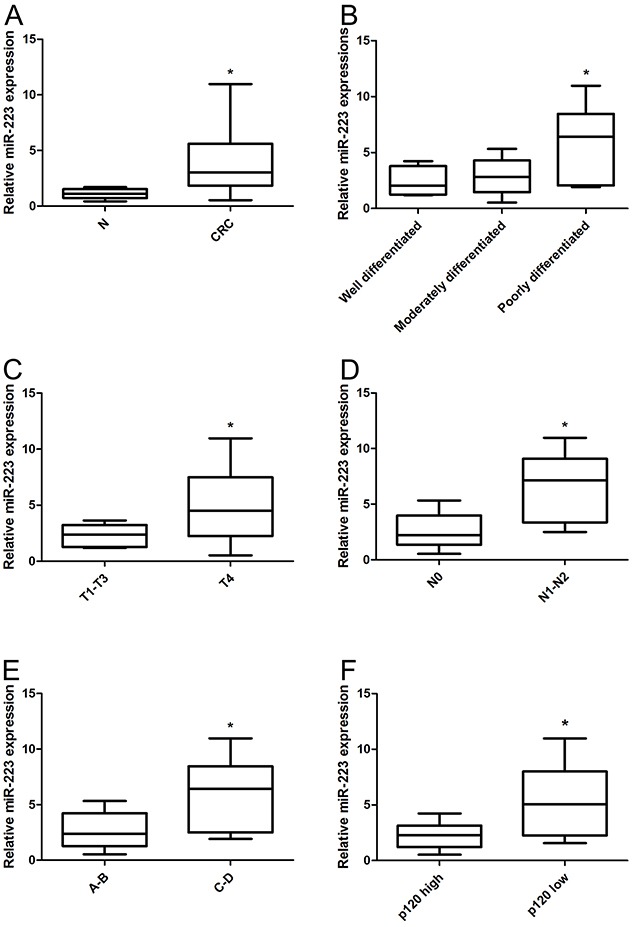
miR-223 is upregulated in human colon cancer tissues Total RNA was extracted from 18 colon cancer tissues and corresponding normal tissues, and miR-223 was detected by real-time PCR. miR-223 was upregulated in colon cancer tissues compared with normal colon **(A)**, miR-223 upregulation is associated with colon cancer differentiation **(B)**, invasion **(C)**, lymph node metastasis **(D)**, and Dukes stage **(E)**. miR-223 was upregulated in p120 low expression tissues compared with p120 high expression tissues **(F)**. n=3; *P<0.05 vs. normal control.

Real-time PCR showed that miR-223 expression in colon cancer tissues was higher than in normal colon tissues (P<0.05) (Figure 2A). miR-223 expression in poorly differentiated carcinoma was higher than in moderately and well differentiated carcinoma (P<0.05). Expression in the T4 stage was higher than in T1–T3 (P<0.05); similar results were obtained in tumors with different lymph node statuses (P<0.05) and different Dukes stages (P<0.05) (Figure 2B-2E). In colon cancer tissues, p120 expression was negatively correlated with miR-223 expression (r=-0.575, P=0.013) (Figure 2F) (Table 3).

**Table 3:** p120 and miR-223 correlation analysis in colon cancer

		miR-223 expression^#^		*r*	*P*
		High	Low		
**p120 expression**	**Low**	7	3		
	**High**	1	7	−0.575	0.013*

^#^miR-223 levels higher than the median value were considered high expression; levels lower than the median value were considered low expression.

*P<0.05.

### p120 is a direct target of miR-223

**Figure 3:**
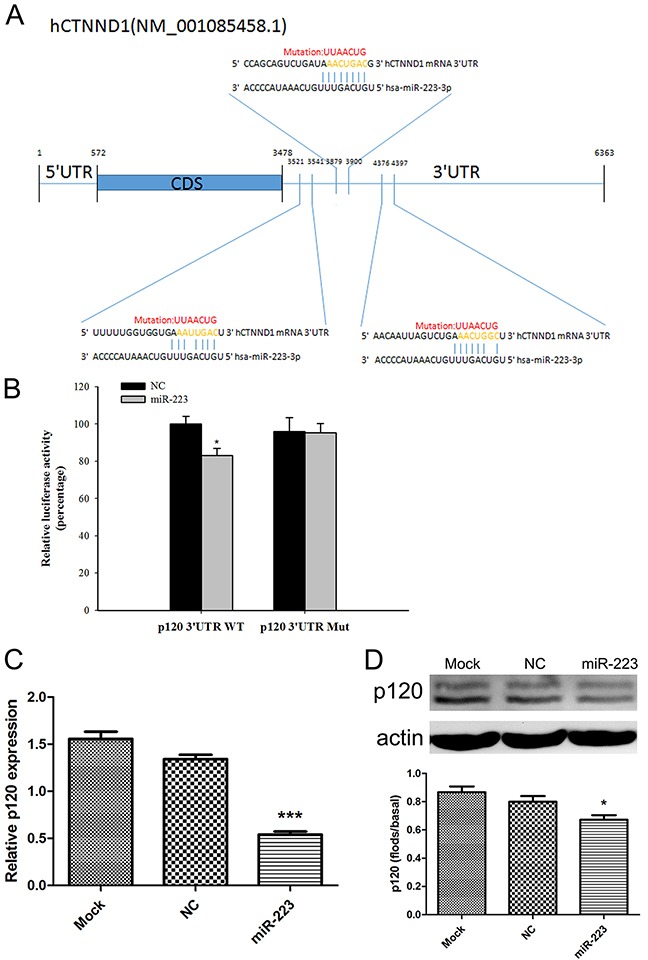
miR-223 directly targets p120 Mutation site map: wild-type p120 (p120-3′UTR WT plasmid) contains a sequence capable of binding miR-223, and a mutated plasmid (p120-3′UTR Mut) was created by mutating the 414, 55, and 911 sites of the p120 mRNA 3′UTR **(A)**. Relative luciferase activity was measured after HEK293 cell co-transfection with miR-223 mimics (or negative control) and wild-type (or mutant) plasmids **(B)**. LoVo cells were transfected with miR-223 mimics or negative control; 24 h later, p120 was detected via real-time PCR **(C)**. 48 h after transfection, p120 was detected by western blotting, with actin as the loading control **(D)**. Representative images shown are from independent experiments repeated three times. The bottom bar graphs are quantified results. n=3; ***P<0.001, *P<0.05 vs. the control group.

The bioinformatics databases TargetScan and MicroRNA.org predicted that the miR-223 5′ seed region may complementarily bind with the p120 mRNA 3′ UTR region. To confirm this direct interaction, we performed the dual luciferase reporter assay. Luciferase activity was decreased in the p120-3′UTR WT plasmid group (P<0.05), but was the same in the p120-3 'UTR Mut plasmid group compared with the negative control (P>0.05) (Figure 3A, 3B). Real-time PCR showed that p120 expression was decreased in LoVo cells transfected with miR-223 mimics (P<0.001) (Figure 3C). p120 protein was also decreased compared to negative controls (P<0.05) (Figure 3D).

### miR-223 overexpression enhances LoVo cell proliferation and regeneration

**Figure 4:**
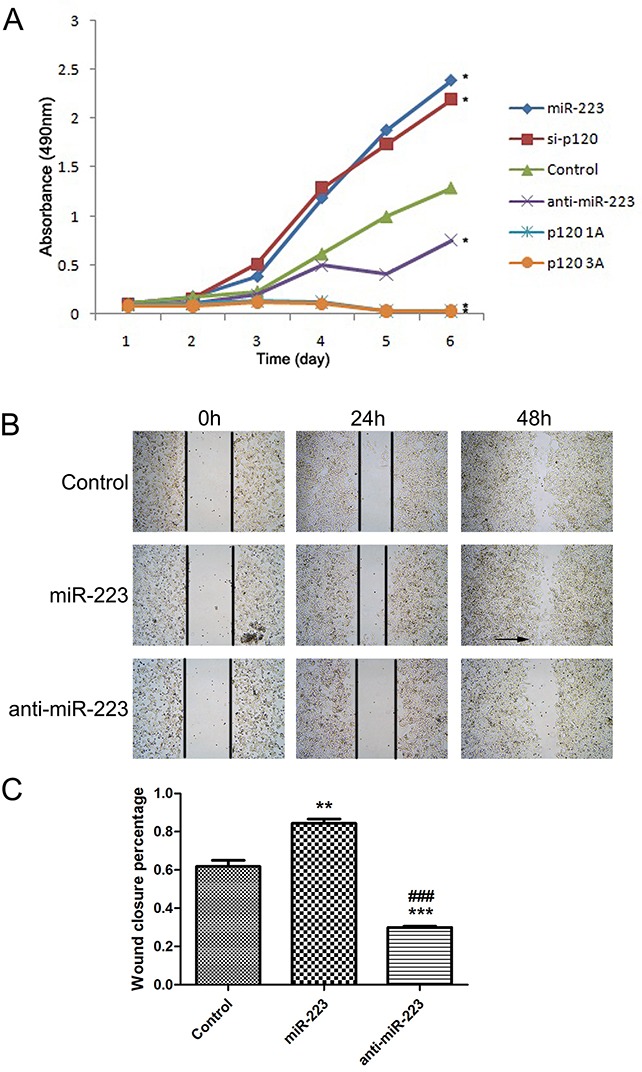
miR-223 overexpression enhances LoVo cell proliferation and regeneration MTT assay was used to detect transfected LoVo cell proliferation **(A)**. Results were averaged over three independent experiments, with three parallel wells per experiment. LoVo cells were transfected with miR-223 mimics, miR-223 inhibitor, or negative control, and a wound-healing assay was performed **(B)**. Arrows show that cells on both sides of the wound were fused. Representative images shown are from independent experiments repeated three times. 48 h after the scratch, wound closure percentage was quantified **(C)**. n=3; *P<0.05, **P<0.01, ***P<0.001 vs. the control; ###*P*<0.001 vs. miR-223 group.

We employed synthesized miR-223 mimics or an inhibitor (anti-miR-223) to up- or downregulate miR-223 expression in LoVo cells. MTT results showed that cell proliferation increased in LoVo cells transfected with miR-223 mimics, and decreased in inhibitor-transfected cells compared with the negative control (Figure 4A). A wound-healing assay showed that 48 h after scratching, cells transfected with miR-223 mimics re-attained confluency more quickly than miR-223 inhibitor-transfected cells compared with the control (Figure 4B, 4C).

### miR-223 overexpression enhances LoVo cell migration and invasion

**Figure 5:**
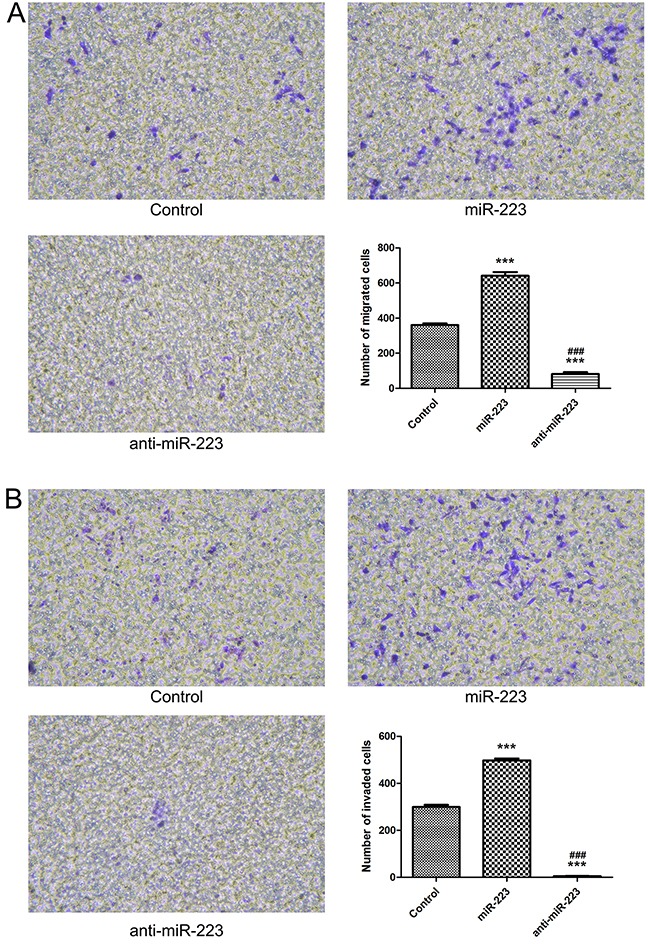
miR-223 overexpression enhances LoVo cell migration and invasion LoVo cells were transfected with miR-223 mimics, miR-223 inhibitor, or negative control. Transwell cell migration assay **(A)**. Matrix gel invasion assay **(B)**. n=3; ***P<0.001 vs. the control group; ### *P*<0.001 vs. the miR-223 group.

A transwell cell migration assay showed increased numbers of migrated cells in the miR-223 mimics group, and reduced numbers in the miR-223 inhibitor group compared with the control (Figure 5A). A Matrigel invasion assay showed similar results, with higher invaded cell numbers in the miR-223 mimics group, and lower numbers in the miR-223 inhibitor group compared with the control (Figure 5B). These results demonstrated that miR-223 promotes a malignant phenotype in human colon cancer cells.

### miR-223 overexpression recapitulated epithelial-mesenchymal transition induction in LoVo cells

**Figure 6:**
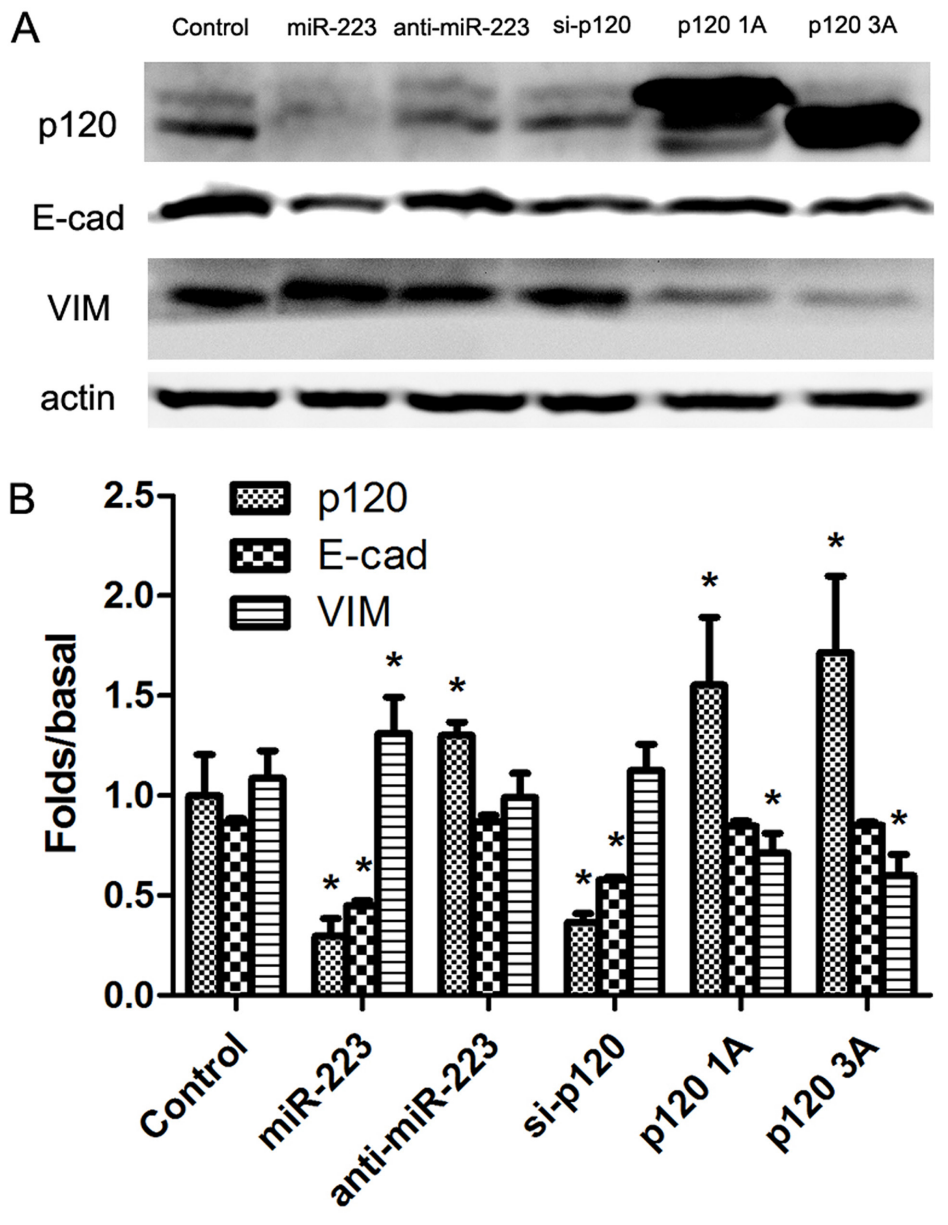
miR-223 overexpression recapitulated EMT induction in LoVo cells LoVo cells were transiently transfected with miR-223 mimics, miR-223 inhibitor, or negative control, respectively, and were transfected with p120 siRNA, p120 1A or p120 3A plasmid as control. 48 h after transfection, p120, E-cad, and VIM were detected via western blotting, with actin as the loading control **(A)**. Representative images shown are from independent experiments repeated three times. Quantified western blotting results **(B)**. n=3; *P<0.05 vs. the control group.

We hypothesize that miR-223 oncogenic functions may be mediated by direct targeting and negatively regulation of p120. p120 maintains the cell adhesion and regulates E-cad stability. Western blotting showed that p120 and E-cad were decreased in miR-223 mimic-transfected cells, whereas VIM expression increased. Transfection with miR-223 inhibitor increased p120 expression, but E-cad and VIM levels were unchanged. p120 and E-cad were downregulated in the p120 siRNA group, while VIM was unchanged. In the p120 1A and 3A transfection groups, p120 1A and 3A levels increased, E-cad was unchanged, and VIM was downregulated (Figure 6A, 6B). These results suggest that miR-223 overexpression may induce EMT, possibly by mediating p120.

### miR-223 overexpression increases nuclear expression of β-catenin in LoVo cells

**Figure 7:**
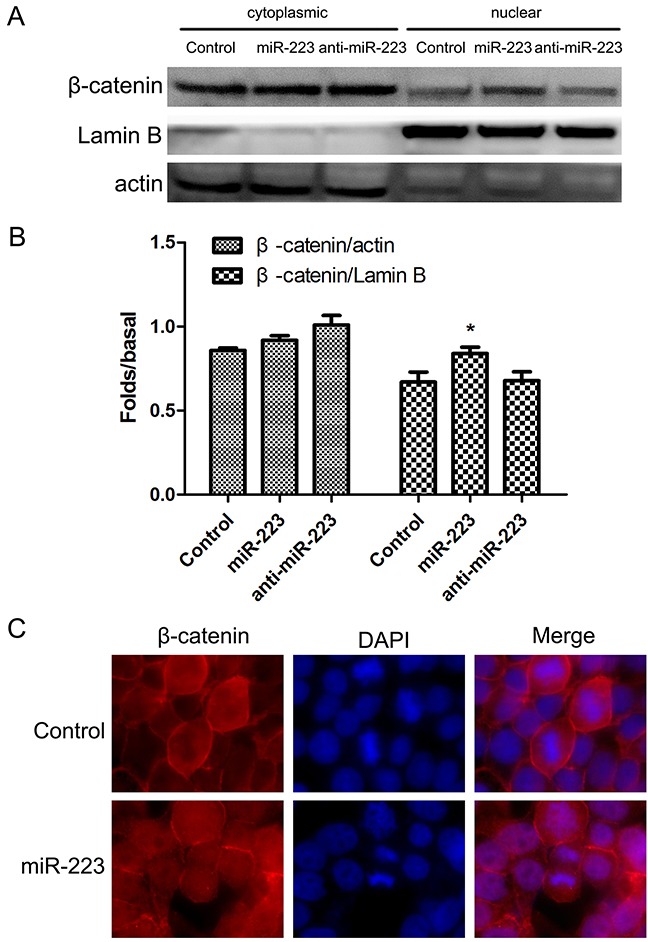
miR-223 overexpression increases nuclear β-catenin in LoVo cells LoVo cells were transfected with miR-223 mimics, miR-223 inhibitor, or negative control for 48 h, and cytoplasmic and nuclear β-catenin were detected by western blotting **(A)**. Actin and lamin B were used as cytoplasmic and nuclear protein loading controls, respectively. Representative images shown are from independent experiments repeated three times. Quantified western blotting results **(B)**. n=3; *P<0.05 vs. the control group. LoVo cells were transfected with miR-223 or negative control for 24 h, fixed with 4% paraformaldehyde, and fluorescently stained for β-catenin. DAPI was used to stain nuclei **(C)**. At least five fields of view were imaged using a laser confocal microscope under each condition.

We demonstrated that overexpression of miR-223 mimics reduced p120 and E-cad levels on the cell membrane. We hypothesized that this E-cad downregulation may result in β-catenin (from the E-cad/p120/β-catenin complex) accumulation in the cytoplasm and translocation into the nucleus. β-catenin can reportedly be transposed into the nucleus after aggregation in the cytoplasm, activating transcription of TCF/LEF signal and related target genes. We found that transfection of miR-223 mimics increased nuclear expression of β-catenin (Figure 7A, 7B), which was confirmed by immunofluorescent staining (Figure 7C).

### miR-223 overexpression upregulated c-Myc, cyclinD1, and MMP7 in LoVo cells

**Figure 8:**
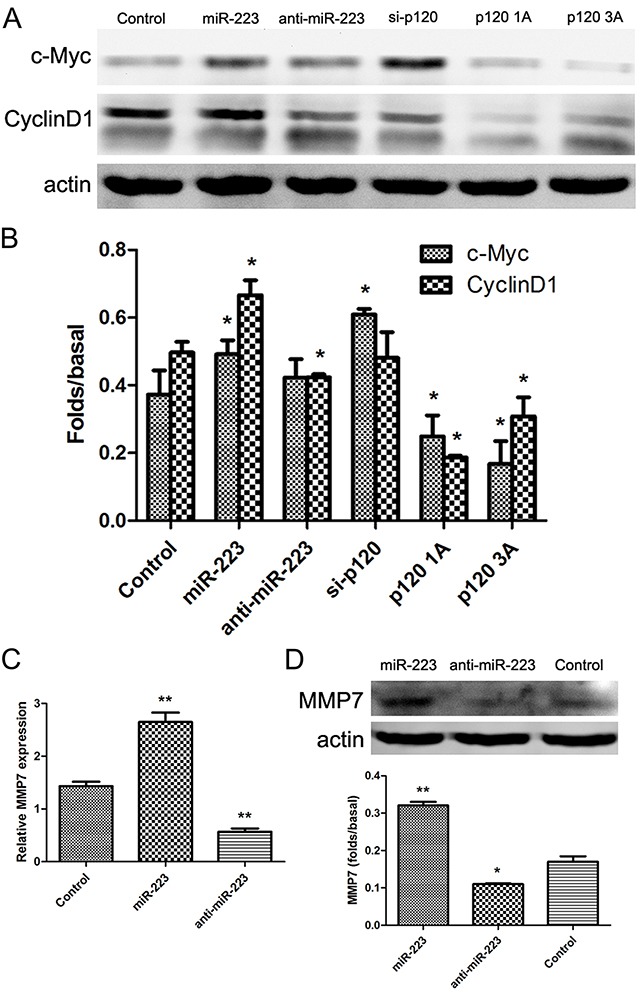
miR-223 overexpression upregulates c-Myc, cyclinD1, and MMP7 in LoVo cells LoVo cells were transiently transfected with miR-223 mimics, miR-223 inhibitor, or negative control, respectively, and were transfected with p120 siRNA, p120 1A, or p120 3A plasmid as control. 48 h after transfection, c-Myc and cyclinD1 were detected by western blotting, with actin as the loading control **(A)**. Quantified western blotting results **(B)**. LoVo cells were transfected with miR-223 mimics, miR-223 inhibitors, or negative control. 24 h later, MMP7 expression was detected by real-time PCR **(C)**. 48 h after transfection, MMP7 was detected by western blotting, with actin as the loading control **(D)**. Representative images shown are from independent experiments repeated three times. The bottom bar graphs are quantified results. n=3; *P<0.05, **P<0.01 vs. the control group.

We examined expression of c-Myc, cyclinD1, and MMP7, which are downstream of β-catenin and TCF/LEF, and are related to cell proliferation and invasion. Western blot and real-time PCR results showed that c-Myc and cyclinD1 protein levels, and both mRNA and protein levels of MMP7, increased in the miR-223 mimics group (Figure 8A-8D).

### miR-223 overexpression enhances RhoA activity in LoVo cells

**Figure 9:**
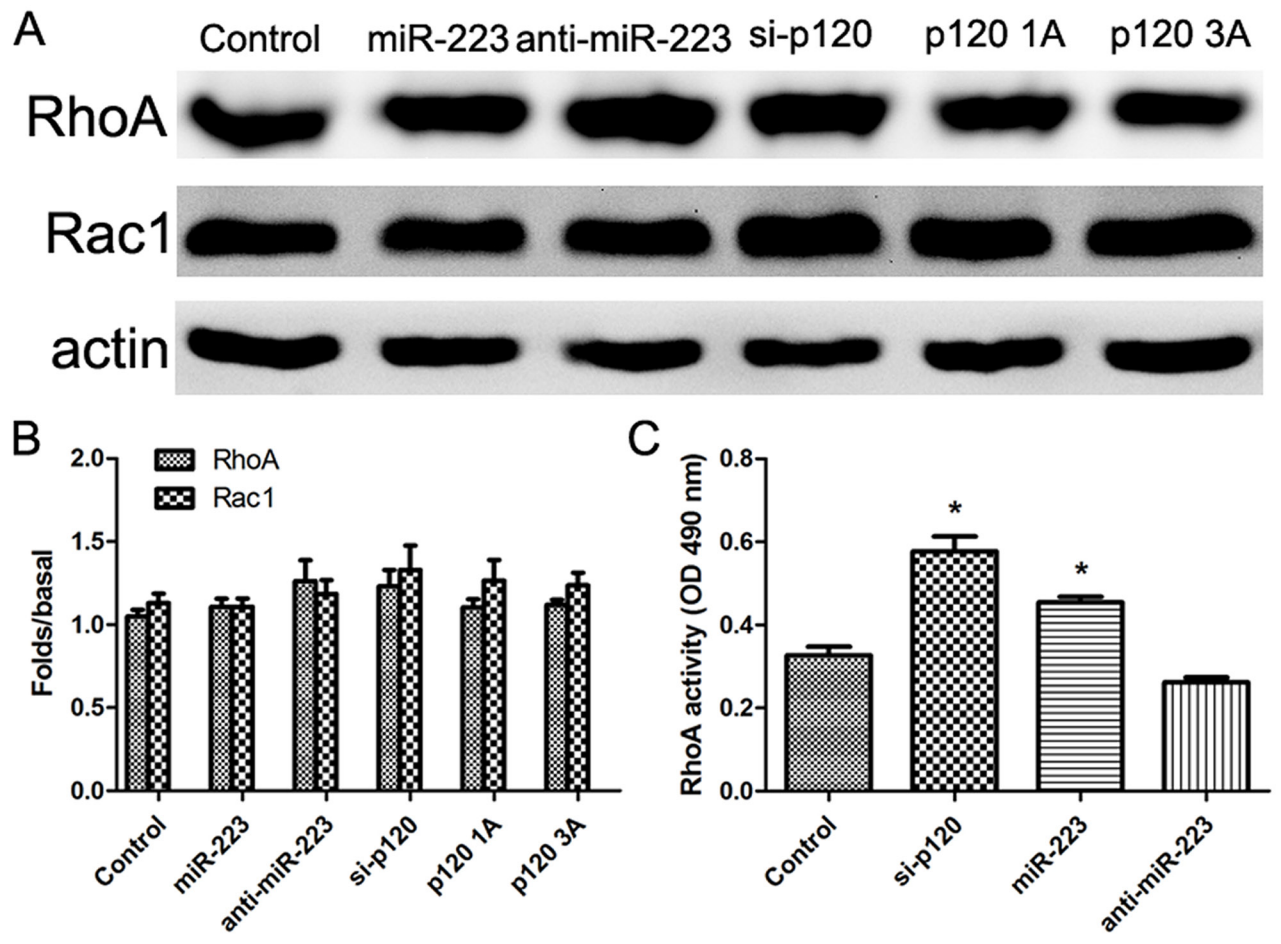
miR-223 overexpression enhances RhoA activity in LoVo cells LoVo cells were transiently transfected with miR-223 mimics, miR-223 inhibitor, or negative control, respectively, and were transfected with p120 siRNA, p120 1A, or p120 3A plasmid as control. 48 h after transfection, RhoA and Rac1 were detected by western blot, with actin as the loading control **(A)**. Representative images shown are from independent experiments repeated three times. Quantified western blotting results **(B)**. LoVo cells were transiently transfected with miR-223 mimics, miR-223 inhibitor, negative control, or p120 siRNA. The activity of RhoA was detected by G-LISA **(C)**. n=3; *P<0.05 vs. the control group.

Rho GTPase family members, RhoA and Rac1, play roles in cell migration. Western blotting results showed that RhoA and Rac1 levels remained the same in miR-223 mimics, miR-223 inhibitor, p120 siRNA, p120 1A, or p120 3A transfected cells (Figure 9A, 9B). However, G-LISA results showed that RhoA activity was increased in the miR-223 mimics and p120 siRNA transfection groups (Figure 9C).

## DISCUSSION

miR-223 can act as a tumor suppressor or an oncogene in different environments. Li, *et al*. [20] found that miR-223 overexpression in esophageal cancer cells decreased cell migration and invasion by targeting artemin (ARTN), and thus acted as a tumor suppressor. Similarly, Jia, *et al*. [21] indicated that by targeting insulin-like growth factor 1 receptor (IGF-1R), miR-223 inhibited hepatoma cell proliferation. Conversely, Kurashige, *et al*. [22] showed that miR-223 was overexpressed in patients with esophageal squamous cell carcinoma, targeting F-box/WD repeat-containing protein 7 (FBXW7) and acting as an oncogene. In gastric cancer, miR-223 promoted invasion and metastasis by targeting the tumor suppressor, EPB41L3 erythrocyte membrane protein band 4.1 like 3 (EPB41L3) [23]. The present study found that miR-223 expression was increased in colon cancer, most significantly in advanced stage tumors with poor differentiation, enhanced invasion, and lymph node metastasis. This suggests that miR-223 is an oncogene in colon cancer, consistent with previous findings [18].

We also found that p120 and miR-223 levels were negatively correlated in colon cancer clinical specimens. miRNAs negatively regulate target genes by binding complementary (or nearly-so) mRNA sequences to promote target decay or inhibit target mRNA translation. Both the TargetScan and MicroRNA.org databases predicted that the miR-223 5′ seed region may complementarily bind to the p120 mRNA 3′UTR region. Using the PITA algorithm, we found that the miR-223 5′ seed region contains eight nucleotides fully complementary to the p120 mRNA 3′UTR in three regions (numbers 414, 55, and 911 of the p120 mRNA 3′UTR). Therefore, we predicted that miR-223 directly targets p120. Based on the 3′UTR construct of wild-type p120 mRNA, a mutant construct with all the three regions (414, 55, 911) mutated which can disrupt the interaction with miR-223 was generated. Dual luciferase reporter assay results showed decreased luciferase activity in the wild-type p120 mRNA 3′UTR group compared with the mutant construct when cells were co-transfected with miR-223 mimics. Furthermore, overexpressing miR-223 in LoVo cells downregulated p120. These results indicate that miR-223 directly bound the p120 mRNA 3′UTR.

p120 was initially identified as a Src substrate [24] and is now known to mediate cell adhesion through dynamic regulation of the actin cytoskeleton, transportation of cadherin to the cell membrane, and stabilization of cadherin at the cell membrane [25]. p120 downregulation reduces this cadherin stability, leading to cadherin degradation, impaired cell-cell adhesion, increased cell migration and invasion, and ultimately tumor progression [26]. We observed p120 downregulation in colon cancer tissues, and low p120 expression was associated with poor tumor differentiation, lymph node metastasis, and advanced colon cancer stages. These results are consistent with previous findings [11, 27], and emphasize not only the importance of p120 in cell adhesion maintenance, but also its tumor suppressor effect. When the transverse colon was defined as the right side colon, p120 downregulation was more common in patients with right side colon cancer, which may partly explain why those patients whose primary tumors originated on the left side of the colon survive longer than those whose tumors originate on the right side [28]. We speculate that during colon cancer development, miR-223 may target tumor suppressor gene *p120*, negatively regulating *p120* expression, and promoting colon cancer cell invasion and metastasis.

Our study found that in LoVo cells, miR-223 overexpression promotes cell proliferation, migration, and invasion, and miR-223 silencing can inhibit these effects. This result is consistent with that of Zhang, *et al*. [18], and supports our conclusion that miR-223 plays an oncogenic role in colon cancer. miR-223 overexpression in LoVo cells also downregulated p120 and E-cad, and upregulated VIM. We also found that siRNA-mediated p120 silencing in LoVo cells reduced E-cad and increased VIM expression, recapitulating the invasive phenotype. p120 1A or p120 3A plasmid expression in these cells may reduce VIM and partially increase E-cad expression. These results further confirm that miR-223 directly targets p120.

Abnormal β-catenin distribution in cells correlates with tumor progression and p120 expression [29–31]. Kaulfu, *et al*. [32] silenced p120 in PC-3 prostate cancer cells and found that β-catenin accumulates in the nucleus due to reduced p120 expression at the cell membrane. Perez-Moreno, *et al*. [30] showed that cadherin-catenin complex destruction is associated with β-catenin release from the AJ and translocation into the nucleus. Immunofluorescent staining and western blotting of cytoplasmic and nuclear extracts found that in LoVo cells overexpressing miR-223, β-catenin expression increased in the nucleus, which may possibly be associated with miR-223 targeting p120. β-catenin is a component of the Wnt signaling pathway, and its translocation into the nucleus can activate TCF/LEF transcription factors to induce expression of a large number of genes related to cell proliferation, migration, and invasion [33]. Our study found that miR-223 overexpression upregulated the β-catenin downstream signaling molecules, c-Myc, cyclinD1, and MMP7, which are associated with cell proliferation and invasion.

Tumor cell migration also plays a critical role in metastasis, and is a complex process involving cytoskeletal changes and precise control of cell adhesion. Cell adhesion is reduced by p120 and E-cad downregulation in adhesion complexes, and the RhoGTP enzyme family mediates changes in the cytoskeleton [34]. In LoVo cells co-transfected with miR-223 mimics or inhibitor, and either p120 siRNA or p120 1A and 3A plasmid, RhoGTP enzyme family members RhoA and Rac1 were not affected. However G-LISA results showed increased RhoA activity in cells overexpressing miR-223 or with p120 silenced. RhoA switches between a GTP-binding active form and GDP-binding inactive form [35, 36]. When binding GTP, RhoA can interact with downstream effector molecules and promote signaling transduction and cell migration [37]. Thus, miR-223 overexpression may enhance cell migration by inhibiting p120 and increasing RhoA activity.

Wang, *et al*. [38] demonstrated that miR-223 acts as a pro-inflammatory factor in inflammatory bowel disease, a premalignant lesion that can lead to colon cancer, by targeting claudin 8 (CLDN8). Our previous study demonstrated that reduced p120 expression causes inflammation by activating RhoA and regulating NF-κB signaling [39, 40]. Inflammation and cancer are inextricably linked, and abnormal inflammatory responses can initiate tumor development. Inflammatory cells producing cytokines can stimulate cancer cell growth and survival, and p120 deletion can produce an inflammatory tumor microenvironment to promote tumor development [9]. Our results confirm that miR-223 targets p120 and acts as an oncogene in colon cancer, and we hypothesize that miR-223 may regulate the inflammatory response by targeting p120.

## MATERIALS AND METHODS

### Patients and specimens

Formalin-fixed, paraffin-embedded tissue blocks from 80 patients with colon cancer who had undergone colectomy between January 2013 and May 2015 at Tongji Hospital, Huazhong University of Science and Technology, Tongji Medical College, were selected from the archives. Patients included 47 males and 33 females, aged 23–90 years (median age, 58). Additionally, 42 normal colon tissue samples were obtained via surgical resection at distal margin sites. The criteria of patients we chose is the diagnosis of colon cancer and received neither chemotherapy nor radiotherapy before surgery, and this study was approved by the Hospital's Ethics Committee.

### Immunohistochemistry

Both colon cancer and normal tissue blocks were cut into 4 μm-thick sections and mounted on glass slides. IHC staining was performed using the SP two-step method. Antigens were retrieved in preheated 0.1 M citrate buffer (pH 6.0) using a pressure cooker at 90 kpa for 90 sec. Endogenous peroxidase activity was then blocked using 3% hydrogen peroxide at room temperature for 10 min, and nonspecific antigens were blocked with 2% bovine serum albumin (BSA, Sigma, St Louis, MO, USA) in TBS for 1 h. Sections were then incubated with p120 polyclonal goat anti-mouse antibody (1:200) overnight at 4°C, and then secondary antibody (Polink-2 plus® Polymer HRP Detection System) (Beijing Zhongshan Golden Bridge Biotechnology, PV-9003 anti-goat) at room temperature for 30 min. Signal was visualized using the Liquid DAB Substrate Kit. PBS was used as a primary antibody negative control.

### Evaluation of immunostaining

IHC staining results were assessed by two pathology professors and a consensus regarding controversial cases was reached using a multiheaded microscope. p120 is normally expressed on cell membranes in colonic mucosa, and stained brown. The percentage of positive cells was counted and combined with staining intensity to calculate staining score.

Staining score evaluation criteria were as follows [41]: (A) the percentage of positive cells was scored as: no staining for 0, 1–25% for 1, 26–50% for 2, 51–75% for 3, 76–100% for 4; (B) staining intensity was scored as: no staining for 0, weakly for 1, moderately for 2, strong for 3. The final score for each slice was defined as A×B. To facilitate statistical analysis, staining scores of 0–3 were classified as p120 low expression, and 4+ points were classified as p120 high expression.

### Quantitative real-time PCR

Total RNA from colorectal tumors and adjacent normal mucosa was extracted using EASY spin (Aidlab Biotechnologies Co., Ltd), and total RNA from LoVo cells was extracted using TRIzol reagent. Quantitative real-time PCR was performed as described previously [42]. Primer sequences were as follows: U6: F: 5′-CTC GCT TCG GCA GCA CAT A-3′, R: 5′-CGA ATT TGC GTG TCA TCC T-3′; miR-223: F: 5′-GGG GGT GTC AGT TTG TCA AAT-3′, R: 5′-CAG TGC AGG GTC CGA GGT AT-3′; p120: F: 5′-GGA CAC CCT CTG ACC CTC G-3′, R: 5′-GCT TGC TAA ACT TCC TCG CTC-3′; MMP7: F: 5′-CCG CGT CAT AGA AAT AAT GCA GAA-3′, R: 5′-GAT GTC AGC AGT TTC CCA ATC AAC-3′; GAPDH: F: 5′-ACC AGC CCC AGC AAG AGC ACA AG-3′, R: 5′-TTT GCT TGA AGT TTC ACT GGC ATC-3′.

### Dual luciferase reporter assay

HEK-293T cells were seeded in 96-well plates. After 24 h incubation, cells were co-transfected with psiCHECK-p120 3′UTR WT or psiCHECK-p120 3′UTR Mut, and miR-223 mimics or negative control mimics. Forty-eight h after transfection, cells were assayed using the dual luciferase assay system (Promega, Madison, USA) according to the manufacturer's instructions. All transfection experiments were conducted in triplicate and repeated three times independently.

### Colorectal cancer cell culture and transfection

The human colorectal carcinoma cell line, LoVo, was cultured in Dulbecco's modified Eagle's medium (DMEM) supplemented with 10% fetal bovine serum (FBS; Gibco). Artificial miR-223 mimics and an inhibitor were synthesized by GenePharma. After 20 h incubation, LoVo cells were transfected with either miR-223 mimics, anti-miR-223, p120 siRNA, p120 1A, or p120 3A using Lipofectamine 2000 (Invitrogen, Carlsbad, CA, USA) according to the manufacturer's instructions.

### Cell proliferation assay

The effect of miR-223 on cell viability was measured by MTT assay. Cells were plated at 500 cells/well in 96-well plates in triplicate. A standard MTT assay was performed on days 1–6 following transfection. All assays were repeated at least three times.

### Scratch wound-healing assay

Cells were seeded and grown to confluence in 6-well dishes, and transfected with either miR-223 or anti-miR-223. 24 h later, an artificial wound was scratched into the confluent cell monolayer using a 200-μl pipette tip. To visualize cell migration and wound healing, images were captured 0 and 48 h after scratching. The distance between the two edges of the wound was measured using Digimizer software (MedCalc Software, Ostend, Belgium).

### Transwell migration and invasion assays

Cell migration and invasion were assessed using the two-chamber assay with the 8 mm-pore 24-well BD FALCON Cell Culture Inserts and BD BioCoat Matrigel Invasion Chambers (BD Bio sciences, Bedford, MA), respectively. LoVo cells were transfected with miR-223 mimics, anti-miR-223, or negative control and cultured for 12 h. Then, 5×10^5^ cells/chamber were seeded in the upper chamber and cell migration and invasion were assessed 24 h later. Migrated or invaded cells were counted in five randomly selected high power fields.

### Western blot

Cytoplasmic and nuclear protein extraction, and western blot analysis were performed as described previously [39].

### Immunofluorescent analysis

Cells were fixed with 4% paraformaldehyde, permeabilized with 0.3% Triton X-100, and incubated with primary antibodies at 4°C overnight. Antibodies directed against β-catenin were obtained from Cell Signaling Technology (San Jose, CA, USA). Nuclei were stained with DAPI. Cells were photographed using an Eclipse FV500 confocal laser scanning microscopy system (Olympus, Tokyo, Japan) with the appropriate filter sets.

### RhoA activation assay

The RhoA G-LISA® kit was purchased from Cytoskeleton, Inc. (Cat. #BK124; Denver, CO, USA). Transfected LoVo cells were collected in ice-cold lysis buffer, immediately snap frozen in liquid nitrogen, and stored at −80°C to minimize GTP hydrolysis. An aliquot was set aside for protein concentration determination using the Precision Red Advanced Protein Assay Reagent supplied with the kit. Equal protein aliquots were added to the individual wells in eight-well strips supplied with the kit, and the strips were incubated on a cold orbital microplate shaker at 400 rpm at 4°C for exactly 30 min. The strips were washed and incubated with an anti-RhoA primary antibody followed by an HRP-conjugated secondary antibody, and the HRP detection reagent supplied with the kit was used. Absorbance was measured at 490 nm using a microplate spectrophotometer (SpectraMax 340 Microplate Reader; Molecular Devices). Samples from at least three independent experiments were assayed in triplicate.

### Statistical analysis

Results are expressed as means ± SD of experiments repeated at least three independent times. Statistical significance was determined using SPSS 19.0 software (IBM Corporation, New York, USA). Data were evaluated via one-way analysis of variance (ANOVA) combined with a post hoc analysis (Fisher's PLSD). Spearman rank correlation analysis was used to assess correlations between p120 and miR-223 levels in colon cancer. P<0.05 was considered significant.
